# Matrix scaffolding for stem cell guidance toward skeletal muscle tissue engineering

**DOI:** 10.1186/s13018-016-0421-y

**Published:** 2016-07-27

**Authors:** Claudia Fuoco, Lucia Lisa Petrilli, Stefano Cannata, Cesare Gargioli

**Affiliations:** Department of Biology, Rome University Tor Vergata, Rome, Italy

**Keywords:** Extracellular matrix, Skeletal muscle, Tissue engineering, Biomaterials, Biomimetic scaffold

## Abstract

Extracellular matrix (ECM) is composed of many types of fibrous structural proteins and glycosaminoglycans. This important cell component not only provides a support for cells but is also actively involved in cell-cell interaction, proliferation, migration, and differentiation, representing, therefore, no longer only a mere static structural scaffold for cells but rather a dynamic and versatile compartment. This aspect leads to the need for investigating new bio-inspired scaffolds or biomaterials, able to mimic ECM in tissue engineering. This new field of research finds particular employment in skeletal muscle tissue regeneration, due to the inability of this complex tissue to recover volumetric muscle loss (VML), after severe injury. Usually, this is the result of traumatic incidents, tumor ablations, or pathological states that lead to the destruction of a large amount of tissue, including connective tissue and basement membrane. Therefore, skeletal muscle tissue engineering represents a valid alternative to overcome this problem.

Here, we described a series of natural and synthetic biomaterials employed as ECM mimics for their ability to recreate the correct muscle stem cell niche, by promoting myogenic stem cell differentiation and so, positively affecting muscle repair.

## Background

The human body possesses the ability to recover any minor damage while, after an acute injury or an extensive defect caused by disease, congenital malformations, or surgical removal, it is much more arduous or even impossible for the body to heal on its own. This is particularly true for the skeletal muscle that has an endogenous capability to recover injured tissue by activating a response regeneration process that firstly involves mononuclear resident inflammatory cells, such as mast cells and macrophages. Activated by the necrotic myofiber rupture and the consequent release of factors, mononuclear resident inflammatory cells secrete several chemotactic signals (i.e., tumor necrosis factor (TNF)-α and interleukin (IL)-6) to recruit and activate other circulating inflammatory cells, in particular neutrophils, to the damaged site [1]. Neutrophils, in turn, recruit monocytes that, once invaded the injured tissue, begin to differentiate in macrophages that phagocytize necrotic muscle fibers, thus removing cellular debris. Macrophages can be divided into two different subpopulations, M1 pro-inflammatory and M2 pro-regenerative macrophages. The initial wave of the M1s activates muscle progenitor cells while M2 macrophages stimulate their proliferation and differentiation [1, 2]. In particular, the most important resident multipotent stem cells are satellite cells [3, 4]. These normally quiescent cells, in response to damage, become active, proliferate, and finally differentiate into myotubes that directly participate in the muscle regeneration process by fusing with the existing injured myofibers or by forming new ones [5–7]. This is what commonly happens during muscle regeneration; on the other hand, large damage is unable to recover by satellite cells activation and differentiation. Therefore, alternative strategies were developed to regenerate injured skeletal muscle tissues or even to stimulate muscular tissue self-healing ability, by generating a three-dimensional scaffold for grafting. This approach is based on the isolation and culture of myogenic cells, engulfed on natural or synthetic 3D scaffolds acting as the extracellular matrix (ECM), and already demonstrated regulating different cell functions such as survival, migration, proliferation, and differentiation [8, 9].

In this review, we will describe the importance of the ECM in tissue engineering focusing on different natural (i.e., chitosan, silk fibroin, alginate, and agarose) or synthetic biomaterials (i.e., polylactic acid, polycaprolactone, and polyurethanes) that can be used in skeletal muscle engineering to generate 3D artificial tissue structures.

## ECM as a key regulator for cellular activities

The ability to stimuli response is a distinctive characteristic of our life. We are continuously influenced by our surroundings, and from the single cell to the entire organism, we are able to interpret and adequately respond to the different signals that we receive. The ECM has a fundamental role for cell life: survival, motility, and communication [8, 9]. The ECM is the non-cellular part, present within all tissues and organs, stuffing the space around the cells with its complex network of macromolecules, arranged in a unique three-dimensional organization, whose precise composition and structure varies from tissue to tissue, according to its particular functional need [8]. The ECM not only provides a mere structural scaffold for cells, as has been considered for a long time, but also contributes to cellular and organ-level functions in an active manner. Its importance in this direction is confirmed by the fact that ECM is deregulated in many different types of diseases, such as osteogenesis imperfecta and osteoporosis [10]. The ECM active role is carried out by modulating many cellular functions in different ways, likewise mechanical stimulation, which is achieved through varying the degree of stiffness of the matrix components, directly influencing cell differentiation [11]. This was observed in mesenchymal stem cells (MSCs) able to differentiate into different lineages depending on the substrate stiffness on which they were plated [12]. Moreover, the ECM, by binding many soluble factors (i.e., Wnt proteins, bone morphogenetic proteins (BMPs)) with its components, can store them, so regulating their availability and activity. Furthermore, its proteins can also interact with cell adhesion molecules, influencing the chemical and intracellular signaling and even stem cell differentiation [10]. Therefore, through different stimuli, the ECM affects many cellular processes such as cell growth, adhesion, migration, differentiation, and even gene expression remodeling, not only during the developmental and homeostasis processes but also in response to physiological stress or injury and disease [8, 10]. Moreover, it is important to mention that the ECM is even involved in cell death. In fact, a programmed cell death mechanism, called *anoikis*, exists and is induced by the detachment of cells from the ECM, therefore causing the loss of cell-matrix interactions [13]. Even if the macroscopic arrangement of the ECM does not change during these cell-matrix interactions, the ECM is dynamically remodeled by cells, thanks to the activity of matrix-degrading enzymes such as cathepsins, heparanase, hyaluronidases, and metalloproteases (MMPs), in normal tissue turnover and in disease too [14].

## The main composition of the ECM

There are a large variety of macromolecules that contribute to the ECM structure and function, and they can be primarily divided into different main groups: fibrous ECM proteins, including collagen, elastin, fibronectin, and laminin; proteoglycans (PGs), and glycosaminoglycans (GAGs) [8]. Collagen represents 30 % of the total proteins in humans and is the most ubiquitous and abundant ECM fibrous protein, designed to provide strength and resiliency to tissues, and able to regulate cell adhesion, chemotaxis, and migration, and to guide tissue development [9, 15]. In a given tissue, collagen fibers are generally a heterogeneous mix of different types, even if type I collagen usually predominates, representing the most abundant ECM protein in many adult tissues, such as dermis, bone, and tendon [8, 16]. Collagen synthesis and deposition is mainly related to the activity of fibroblasts. These interstitial cells, residing in the *stroma* or recruited from neighboring tissue upon an injury, are able to organize collagen fibrils into sheets by exerting tension on the matrix, hence influencing the alignment of collagen fibers [10, 17]. Collagen is correlated with elastin, another ECM fibrous component and insoluble protein polymer, synthesized from the precursor tropoelastin, a linear polypeptide of 60–70 kDa. Elastin, together with glycoprotein microfibrils, makes up the elastic fibers of large structures that supply rebound to tissues commonly subjected to repeated stretching forces, such as the lung, heart, large elastic blood vessels, bladder, and elastic cartilage [8, 9]. Fibronectin (FN) is the third fibrous ECM molecule and consists of a protein dimer, composed of two monomers linked by a pair of disulfide bonds, ubiquitously present in the ECMs of different cell types with a crucial role in the ECM organization and then cell attachment and function [9]. FN is involved in cell migration during development and has also been related to different pathological conditions, such as cardiovascular disease and tumor metastasis [15, 18]. Laminins are high molecular weight heterotrimeric proteins composed by an α-chain, a β-chain, and a γ-chain, each of which is encoded by individual genes [19]. Laminins interact with each other, with other ECM molecules and also with resident cells influencing cell adhesion, proliferation, migration, and differentiation, being fundamental for the sustainment and the survival of the tissue [8]. PGs form the basis of ECM structure order and are characterized by a core protein covalently attached with one or more, equal or different, chains of highly negatively charged heteropolysaccharides, called GAGs, such as chondroitin sulfate, dermatan sulfate, keratan sulfate, or hyaluronic acid [8]. PGs can interact with growth factors, cytokines, and chemokines, participating in different cell functional processes, and with other ECM molecules, thereby contributing to the assembly of the ECM scaffold [8, 20].

## Exploiting ECM task for tissue engineering

The involvement of the cell-ECM interactions, in physiological conditions and during regeneration processes induced by an injury or a disease, has been well documented and ascertained during these last two decades. The pivotal role of the ECM led to the conceiving and later the realization of functional substitutes for damaged or diseased tissues or organs, exploiting precisely that influence which ECM exerts on cells and guiding the way to an emerging field, namely tissue engineering. This new interdisciplinary scientific approach, that fuses engineering principles with biological ones, looks at reproducing neo-organogenesis in order to generate functional *ex-vivo* living tissue, combining stem cells and even growth factors with appropriate natural and/or synthetic biomaterials mimicking native ECMs [21]. To do so, biomaterials have to reproduce the biological and mechanical properties of ECMs shaming cell’s native microenvironment and providing a temporary 3D structure in which cells can adhere, grow, differentiate, and organize themselves into a specific spatial arrangement, that is to say, new tissue. Their “bio”-characteristics, such as biocompatibility, biodegradability, and bioresorbability, play a fundamental role, since they have to provide their progressive replacement with newly formed healthy tissue without inducing any inflammatory responses that, on the contrary, could lead to rejection or *necrosis* meantime ensuring the correct functional development of the newly formed tissue [22]. In order to attain this purpose, stem cells combined with ad hoc biomimetic matrix scaffold can be guided to differentiate into the desired cellular type, according to the necessity. This close tie has been highlighted by early studies showing that ECMs could influence in vivo stem cell differentiation and also stemness maintenance, by creating a local microenvironment, so called “niche,” in which stem cells are embedded [15, 23].

## Skeletal muscle tissue engineering hits and failures

Skeletal muscle tissue engineering is challenging being formed by parallel aligned multinucleated syncytia, enclosed in extracellular connective tissue, that have to be reproduced to ensure the appropriate mechanical tissue properties and then functionalities. Myofibers must be vascularized, to guarantee an adequate oxygen supply and transport of nutrients for cellular survival; moreover, they must also be connected to the nervous system and other host tissues, including tendons and neighboring muscles. In looking at the ECM, each muscle fiber is enclosed by the basal lamina, essential in guiding the regenerative process, typical of the skeletal muscle. Moreover, the presence of sulfate GAGs, PGs, and type I collagen also contribute to maintain the structural muscle integrity [25]. Therefore, all the structural and functional peculiarities mentioned above have to be considered for skeletal muscle tissue engineering to succeed. The particular interest in building an artificial functional muscle, replying the natural one, comes from the fact that the skeletal muscle tissue has an endogenous ability to regenerate after a minor damage, mostly thanks to the activation of satellite cells [26]. If, however, severe damage happens, the skeletal muscle tissue is unable to self-repair. For this reason, injuries deriving from massive trauma such as accidents, combat missions, surgical procedures (i.e., aggressive tumor removal) or muscle devastating alterations (i.e., muscular dystrophies) can affect large volumes of muscles leading to the well-known volumetric muscle loss (VML). In the condition in which given the fact that the muscular tissue is unable to regenerate, it is irreversibly substituted by a non-contractile collagen fibrotic scar tissue with a consequent loss of the muscle function [26–28]. A used standardized protocol, representing the best procedure for severe muscle injuries, forecasts the substitution of the wasted tissue with a surgically removed uninjured muscle one, called “muscle flap” [29–32]. Even if it is widely used, this autologous transplant is very invasive and does not avoid the formation of scar tissue [33, 34]. Moreover, the muscular wound size restoration is limited by donor site availability, since skeletal muscle tissues and other approaches must be thought for treatment of VML. The tissue engineering approach for recovering skeletal muscle defects is based on two classical strategies: in vitro and in vivo tissue engineering [35, 36]. The first approach aims at developing mature and functional artificial muscle structures by culturing cells with myogenic potential into a biomimetic matrix until the correct tissue to be implanted on the recipient is formed [30]. On the other hand, the in vivo strategy is based on transplanting cells, alone or in combination with a biomaterial, to reproduce a local niche at the site of damage in order to influence and promote muscle regeneration or reconstruction [30, 37]. A more innovative and promising strategy for skeletal muscle tissue engineering is the in situ approach that is based on the employment of biomaterial as guidance for endogenous regeneration of injured tissue [30]. In general, these three strategies, although differing for benefits and limits, are connected by the necessity to identify good new bio-inspired scaffolds that act as ECM, being able to respond to specific requests such as 3D support, mechanical and physicochemical features supporting myogenic cell differentiation, protecting them from damages induced by immune responses and finally to stimulate vascularization and innervation.

## ECMs in skeletal muscle tissue engineering

A variety of synthetic or natural biomimetic materials were taken into consideration for skeletal muscle tissue engineering with the overall goal of mimicking ECM key properties. Initial muscle organoids were obtained and parallel oriented by employing static stretch into matrigel and native or modified collagen scaffolds, while recently, a more promising native decellularized ECM has been introduced, containing matrix protein and growth factors critical for tissue regeneration [38, 39].

### Decellularized ECMs

In general, decellularized ECMs can be harvested from natural mammalian tissue sources, thanks to the removal of cells and DNA content from native ECM, carried out by employing physicochemical agents, enzymes, detergents, or even a combination of these [40]. These procedures may in fact alter the ECM, compromising its biochemical, mechanical, and structural hallmarks. Hence, optimized decellularization protocols need to be generated basing on the specific requisites. To this purpose, Qing and collaborators proposed as an efficient decellularization protocol the use of a rat skeletal muscle indicating that the muscle must be subjected to an oscillatory treatment at 4 °C with 1 % SDS for 72 h. They showed that the obtained acellular matrices have an intact ECM with the complete removal of muscle fibers [41]. On the other hand, Gillies and colleagues showed that by employing 50 nM latrunculin B for 2 h at 37 °C, hypertonic saline solution (0.6 M potassium chloride for 2 h and 1 M potassium iodide for 2 h), and DNase I 1 kU/ml, it was possible to decellularize the mouse *tibialis anterior* muscle without altering its ECM composition or mechanical properties [42]. Moreover, recently, it has been evaluated the efficiency of enzyme-detergent methods on cell removal of mouse *latissimus dorsi* (LD). Demonstrating that extensive washing of the LD with a mixture of 0.1 % trypsin/0.01 % EDTA for 24 h and 1%Triton X-100 for 1 week could be useful to produce an intact matrix free of cells, showing comparable biomechanical features with the native tissue [43]. Very recently, Badylak’s group developed and characterized the structure, composition and bioactivity, of a perfusion-decellularized porcine *rectus abdominis* (RA) bioscaffold (pM-ECM) showing the ability to support the reconstruction of a partial-thickness abdominal wall alteration in rats. The porcine RA muscle consists purely of cells through continuous perfusion by using a series of chemical and enzymatic treatments via the inferior epigastric artery and vein in a perfusion bioreactor [44]. Decellularized ECMs are considered an ideal candidate scaffold for muscle recovery becoming innervated by the nervous system of the host and to promote new muscle formation, either by activating host cells or by permitting the vehicle of myogenic cells to produce new tissue [45–47]. Some examples of FDA-approved scaffolds are as follows: decellularized ECMs from porcine small intestine submucosa (SIS), human, porcine and bovine dermis, porcine urinary bladder (UB), and different species pericardium and porcine heart valves [48]. The performances of decellularized skeletal muscle ECM (DSM-ECM) implants have shown encouraging results in the repair of skeletal muscle defects. For example, when used in a rat model to rescue hind-limb muscle damage, it was able to recover contractile force measures up to 85 % of pre-injury levels [33]. Interestingly, promising observations were reported from ECM-derived scaffolds, even when they were tested in human patients with muscular deficits carrying out a functional amelioration in three fifths of treated cases [49]. In particular, the ECM-derived scaffold in vivo implantation seems to induce an immune host response that leads to the scaffold degradation, during which the scaffold is re-populated by host-derived mononuclear cells [50, 51]. The degradation of the ECM-derived scaffolds has a positive influence on tissue regeneration since it triggers the release of many bioactive molecules such as vascular endothelial growth factor (VEGF), hepatocyte growth factor (HGF), or insulin-like growth factor (IGF) that chemotactically recruit a variety of cell types, including those capable of myogenesis, to the scaffold implantation site [24, 52–55]. It also seems that these factors directly promote the switch of macrophages from an M1 pro-inflammatory phenotype to an M2 regenerating one [51, 56]. The latter, in combination with the secreted factors, is involved in the activation of stem cells, such as satellite cells, and other progenitor cells, promoting new tissue formation, vascularization, and innervation [57–59].

### Naturally derived ECMs

Other naturally derived molecules have been obtained even from non-mammalian sources [51]. Among them, we can find chitosan, silk fibroin, alginate, and agarose, which are derived from crustacean shells, silkworm cocoons, algae, and seaweed, respectively [60–62]. They have been widely employed in skeletal muscle tissue engineering because they show endogenous bioactive properties and can create complexes with carbohydrates to form molecules, such as ECM heparan sulfate, able to link growth factors [20] [63–68]. For example, alginate has been found that stiffness between 13 and 45 kPa is able to ameliorate myoblast proliferation and differentiation, also promoting the release of a variety of growth factors, such as HGF and VEGF, all important for skeletal muscle regeneration [69–73].

### Synthetically produced ECMs

As mentioned before, the scientific community is even studying the development of synthetic materials that, compared with natural polymers, have the advantage that their mechanical and structural properties can be customized with extreme precision according to the individual needs.

**Fig. 1:**
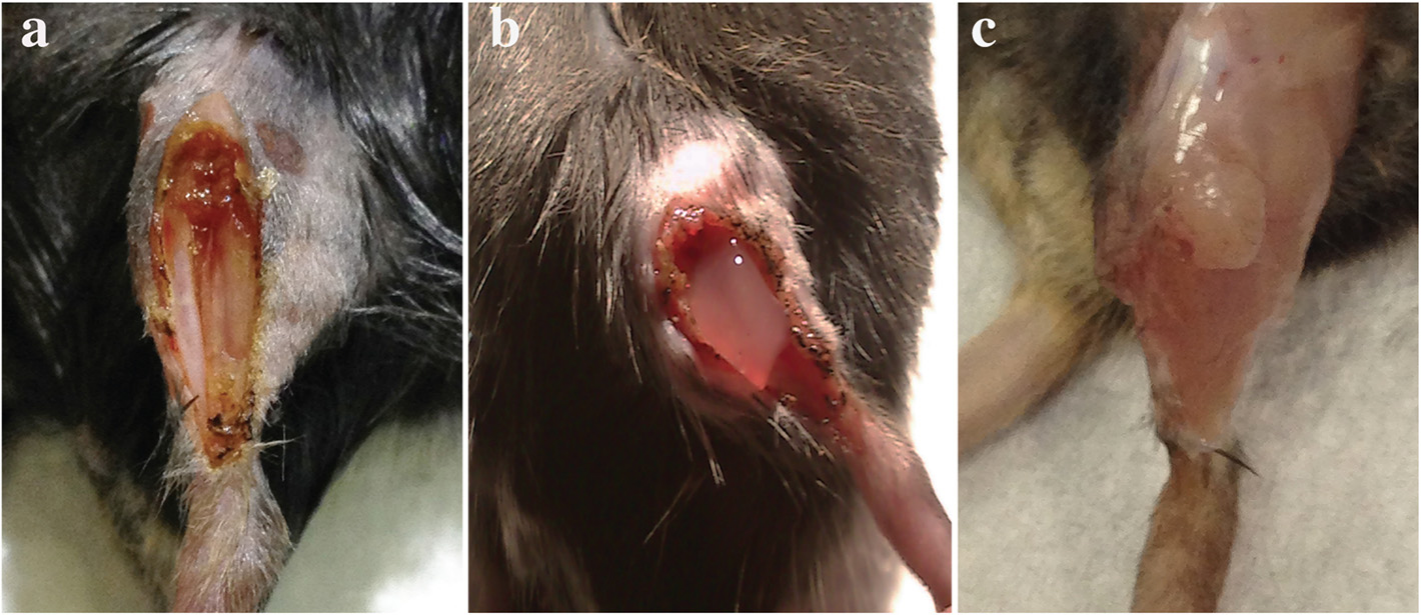
PF-embedded mesoangioblast grafted in an ablated TA lodge, showing full recovery of muscular morphology. **a** Surgical operation dislodging mouse TA. **b** Mesoangioblasts (Mabs) embedded into PF hydrogel scaffold located in the TA lodge. **c** Gross morphology of the TA injury at 40 days after massive muscle ablation, revealing the new artificial TA regeneration when grafted with PF-embedded Mabs

Shandalov and colleagues described a protocol for producing a thick, well-vascularized tissue flap for reconstruction of full thickness abdominal wall defects [71]. Scaffolds were created by mixing poly-l-lactic-acid (PLLA) and polylactic-co-glycolic-acid (PLGA). In particular, a 5 % (*w*/*v*) polymer solution of each was prepared separately, by dissolving 0.5 g polymer in 10 ml chloroform. The two polymers were put together in a 1:1 ratio, to produce a PLLA/PLGA solution. NaCl was sifted to 212–600 μm particles, and 0.4 g was spilled into Teflon cylinder molds and suspended in 0.24 ml PLLA/PLGA solution. Subsequently, chloroform and salt were eliminated, respectively, by evaporation and distilled water washing. The scaffolds were lyophilized and the cells (endothelial cells (ECs), myoblasts, and fibroblasts) suspended in a 1:1 mixture of Matrigel: cell medium ratio was seeded on top of the scaffold. It was cultured in vitro for 10 days to permit the cells to self-assemble, and then, the engineered tissue obtained was bended around the mouse femoral AV veins for another 7 days and, by then well vascularized, was implanted to recover a full thickness abdominal defect [32]. The advantage to use material like this is that they can be scaffolded in many configurations and geometries such as meshes, foams, or hydrogels and they can be even enriched with lithography techniques to create nanoscale patterns which have been demonstrated to improve myoblast alignment and differentiation even in vitro and in vivo [51, 72, 73]. Another important characteristic is that they can be functionalized by chemical modification to improve their performances in regeneration. Moreover, growth factors of other signaling molecules can be incorporated in their bulk structure with the same aim of improving regenerative outcomes. Some promising hybrid materials also exist, such as polyethylene glycol-fibrinogen (PEG-fibrinogen), a hybrid innovative hydrogel formed by pegylated denatured fibrinogen molecules. In particular, the PEG allows to control the material features and the fibrinogen ensures inherent bioactivity, including cell-adhesion motifs and protease degradation sites [74]. This hydrogel has proven applications in three-dimensional cell culture, in cardiac cell therapy and tissue engineering [75]. The ability to undergo controlled and localized liquid-to-solid transition (gelation) in the presence of a cell suspension is one of the main advantages of this biomaterial. This state change can be done both in vitro and also in vivo, for example, inside an injured muscle. Based on this property, we demonstrated the ability of PEG-fibrinogen (PF) as cell carrier to guide and improve the therapeutic effect of donor perivascular myogenic progenitors (mesoangioblasts) [76]. Moreover, by using PF, we were also able to develop in vivo a functional artificial muscle, by encapsulating mesoangioblasts in the bulk of the hydrogel that, once used to replace the mouse ablated *tibialis anterior* (TA), resulted in a muscle tissue practically identical to a physiological one with vascularization and innervation (Fig. 1) [77]. In particular, PF hydrogels were prepared at final concentration of 8 mg/ml diluted with sterile PBS as required. To permit gelation transition, the photo-initiator Igracure™ 2959 was incorporated into PF mixture at the final concentration of 0.1 % *w*/*v* cells which were added at the desired concentration, and 100 μl of the suspension was added into silicon molds and immediately exposed to UV light (365 nm, 4–5 mW/cm^2^) for 5 min. In experiments in which PF was used as carrier, the suspension was conveyed by intra-muscle injection and exposed to UV light (365 nm, 200 mW/cm^2^) using a hand-held light gun for 1 min to allow the photopolymerization reaction. Even if we were able to accurately recreate a vascularized and innervated skeletal muscle, using an innovative methodology that gives the possibility to develop and let mature directly in vivo the artificially created skeletal muscle tissue, we were still far from reaching human-sized tissue, since the scaling up for human clinical therapeutic application represents a crucial goal.

## Conclusions

Biomimetic scaffolds for skeletal muscle tissue engineering are developed considering the ECM not only an implantable medium to vehicle cells and furnish them structural support but also an inductive and bioactive material, able to guide or even substitute endogenous tissue regeneration process.

Here, we have reported some of the purposes and challenges of creating biomimetic scaffolds to support skeletal muscle regeneration after an extensive damage, leading to VML. So, the different natural or synthetic biomaterials described can be used in tissue regeneration, with or without modifications, and mimic the key features of the natural ECM controlling different cell processes such as cell-cell interaction, proliferation, migration, differentiation, cell survival, and even cell death. To date, by using these new approaches, the complete restoration of an extensive damage has not been reached yet; nevertheless, it is evident that those represent a useful instrument deserving further advancement perhaps in combination with other engineering techniques as 3D bio-printing.

## Abbreviations

BMPs, bone morphogenetic proteins; DSM-ECM, decellularized skeletal muscle ECM; ECM, extracellular matrix; FN, fibronectin; GAGs, glycosaminoglycans; HGF, hepatocyte growth factor; IGF, insulin-like growth factor; IL-6, interleukin-6; LD, *latissimus dorsi*; Mabs, mesoangioblasts; MMPs, metalloproteases; MSCs, mesenchymal stem cells; PEG, polyethylene glycol; PF, polyethylene glycol fibrinogen; PGs, proteoglycans; PLGA, polylactic-co-glycolic-acid; pM-ECM, perfusion-decellularized ECM; RA, *rectus abdominis*; SIS, small intestine submucosa; TA, *tibialis anterior*; TNF, tumor necrosis factor; UB, urinary bladder; VEGF, vascular endothelial growth factor; VML, volumetric muscle loss
